# Racial/Ethnic Disparities of Cancer, Metabolic Syndrome, and Lifestyle Behaviors in People under 50: A Cross-Sectional Study of Data from the National Health and Nutrition Examination Survey

**DOI:** 10.3390/epidemiologia3040037

**Published:** 2022-11-03

**Authors:** Lin Zhu, Areebah Rahman, Ming-Chin Yeh, Grace X. Ma

**Affiliations:** 1 Center for Asian Health, Lewis Katz School of Medicine, Temple University, Philadelphia, PA 19140, USA; 2 Department of Urban Health and Population Science, Lewis Katz School of Medicine, Temple University, Philadelphia, PA 19140, USA; 3 Nutrition Program, Hunter College, City University of New York, New York, NY 10065, USA

**Keywords:** metabolic syndrome, cancer, smoking, sleep duration, health disparities

## Abstract

Introduction: Recent epidemiological studies have suggested a trend of increasing prevalence of metabolic syndrome (MetS) and certain types of cancer among adults under age 50. How MetS is associated with cancer in adults under the age of 50, however, remains unclear. Furthermore, it remains unknown whether associations between MetS and cancer vary by racial/ethnic group and whether modifiable lifestyle factors influence MetS–cancer relationships. Methods: We used data from the 2011–2018 National Health and Nutrition Examination Survey (NHANES) to define a case-control sample to examine potential racial/ethnic disparities associated with MetS and cancer of any type. We used a chi-square test and binary logistic regression to examine the MetS and cancer association. Results: From a total sample of 10,220 cases, we identified 9960 no-cancer cases and 260 cancer cases. Binary logistic regression results showed that MetS was significantly associated with a cancer risk among non-Hispanic whites (odds ratio = 1.48, 95% confidence interval = 1.00–2.19); however, it was not associated with a risk among non-Hispanic Blacks, Hispanic/Latinos, or Asian Americans. We also found several significant predictors of cancer, including age, gender, tobacco use, and sleep duration, with their roles varying by racial/ethnic subgroup. Conclusion: The findings of this study indicate that racial/ethnic differences are involved in the association between MetS and cancer, and highlight the potential mediating effects of lifestyle and behavioral factors. Future research should leverage the existing longitudinal data or data from cohort or case-control studies to better examine the causal link between MetS and cancer among racial/ethnic minorities.

## 1. Introduction

Metabolic syndrome (MetS) is a disease entity characterized by a pattern of interconnected physiological, biochemical, and metabolic factors that directly increase the risk of coronary heart disease, other forms of cardiovascular atherosclerotic disease, and type 2 diabetes [1,2,3,4]. These factors include abdominal obesity, insulin resistance, elevated arterial blood pressure, and dyslipidemia. In the International Diabetes Federation (IDF) criteria, MetS is defined with central obesity as a prerequisite plus any two of the four additional factors: raised triglycerides, reduced HDL cholesterol, raised blood pressure, and/or raised fasting plasma glucose [5]. In the past two decades, cumulative evidence has suggested that MetS is an important etiological factor in several types of cancer, including cancer of the liver, breast, prostate, colorectum, and stomach [6,7,8,9,10,11]. Elements of MetS, such as obesity, dyslipidemia, and hyperinsulinemia have been specifically linked to the etiology of these cancer types via cancer-related pathways that regulate cell proliferation, metastasis, and angiogenesis [1,2,4]. Despite the increasing evidence supporting a link between MetS and cancer, three major gaps exist in the current literature.

First, persons under the age of 50 have been understudied in the fields of metabolic health and cancer. Previous studies focused on the general population [6,12,13]. Lifestyle preferences, such as tobacco use and excessive alcohol consumption, as well as environmental and societal factors have played pivotal roles in the rising prevalence of obesity and other cardiometabolic conditions, such as hypertension and hyperlipidemia among younger adults [14,15,16]. The past few decades were also marked by increases in colorectal, pancreatic, and kidney cancer, among other obesity-related cancers [8,17,18,19]. Whether increasing incidence in the two disease categories is related is unknown. It is plausible, however, that the rise in MetS and related components could explain, at least partially, the rise in cancer burden in adults under age 50.

In addition, what is also not well understood regarding the associations between MetS and cancer is the role of race and ethnicity. Compared to non-Hispanic whites, racial/ethnic minority groups are disproportionately affected by various types of cancer and cardiometabolic conditions, including hypertension and diabetes [20,21,22]. The high cancer burden placed on racial/ethnic minorities is attributable to a combination of biological, behavioral, socio-psychological, environmental, cultural, and structural factors [23,24,25]. Hence, literature on the association between MetS and cancer is important, especially since it can shed light on specific approaches to prevention, diagnosis, and treatment in underserved racial/ethnic minority groups.

Furthermore, the roles of modifiable lifestyle behaviors in the MetS–cancer relationship are not well understood. Modifiable lifestyle behaviors, such as physical inactivity, alcohol consumption, tobacco use, and high carbohydrate intake have been associated with an increased risk of metabolic syndrome in studies involving American, Korean, and Taiwanese populations [26,27,28]. However, it remains unclear whether these behaviors act as mediators in the link between MetS and cancer.

The goal of this study was to examine how the association between MetS and cancer might vary across four racial/ethnic groups. We used a nationally representative sample of adults under age 50 and examined the MetS-cancer link, while controlling for various sociodemographic characteristics and modifiable lifestyle behaviors. Findings from this preliminary, cross-sectional study could shed light on future longitudinal studies by helping to better understand the causal relationships between MetS and cancer, and could help guide intervention efforts for disease prevention.

## 2. Materials and Methods

### 2.1. Study Sample

This study is a cross-sectional investigation of adults from the National Health and Nutrition Examination Survey (NHANES) 2011–2018. NHANES is one of a series of health-related programs conducted by the Centers for Disease Control and Prevention’s (CDC) National Center for Health Statistics (NCHS). The objectives of NHANES are to monitor trends in the prevalence of selected diseases and to study the relationship between diet, nutrition, and health [29]. NHANES uses a multistage, stratified design to produce a study sample that is representative of the noninstitutionalized civilian resident population in the 50 states and the District of Columbia [29]. The survey consists of two parts. First, survey questionnaires were administered to eligible participants at home, where person-level demographics, health, and nutrition information were collected. Then, participants were invited to visit specially equipped mobile examination centers for a standardized health examination. The survey procedures are detailed elsewhere [29]. The 2011–2018 NHANES oversampled Asian and several other subpopulations to increase the precision of estimates for these groups. To facilitate the oversampling of the Asian population, NHANES provided survey materials and a promotional video in traditional and simplified Mandarin, Korean, and Vietnamese [30]. This study specifically used a subsample of participants between the ages of 18 and 49. This selection generated a sample of 11,295 individuals. After deleting 1075 cases with missing values on cancer history, the final analysis sample consisted of 10,220 individuals, including 3604 non-Hispanic white, 2382 non-Hispanic Black, 2646 Hispanic/Latino, and 1588 Asian Americans.

### 2.2. Measures

Cancer. Cancer history (yes/no) was based on a self-reported item (“has a physician or health professional ever told you that you have a cancer or malignancy of any kind”?). Participants who responded “yes” were coded as having had cancer.

Metabolic syndrome. To define MetS, we used the 2005 definition from the International Diabetes Federation (IDF) [31]. Under the IDF definition (Table 1), an individual is deemed to have MetS if they have central obesity (waist circumference ≥ 90 cm for South and East Asian men, and ≥80 cm for South and East Asian women, with ethnicity specific values, assumed if BMI is >30 kg/m^2^), plus any two of the following four factors: (1) raised triglycerides (≥150 mg/dL) or specific treatment for this lipid abnormality; (2) reduced high-density lipoprotein (HDL) cholesterol (<40 mg/dL in males, <50 mg/dL in females) or specific treatment for this lipid abnormality; (3) raised blood pressure (blood pressure ≥ 130/85 mm Hg) or treatment of previously identified hypertension; and (4) raised fasting plasma glucose (≥100 mg/dL) or previously diagnosed type 2 diabetes mellitus.

Modifiable lifestyle behaviors. Tobacco use was measured in two categories: whether an individual currently uses any tobacco products or not. In addition, we measured whether an individual was an excessive drinker [32]. Excessive current drinkers reported more than 14 drinks per week on average for men or more than 7 drinks per week on average for women, or 5 or more drinks in a single day at least once in the past year for either sex. We also measured sleep duration in two categories: less than 6 h per day and 6 or more hours per day [33].

Demographic characteristics. We conducted the analysis separately by sex (female or male), age (in years), and education (high school or below; college or some college; or graduate degree).

### 2.3. Statistical Analysis

**Figure 1:**
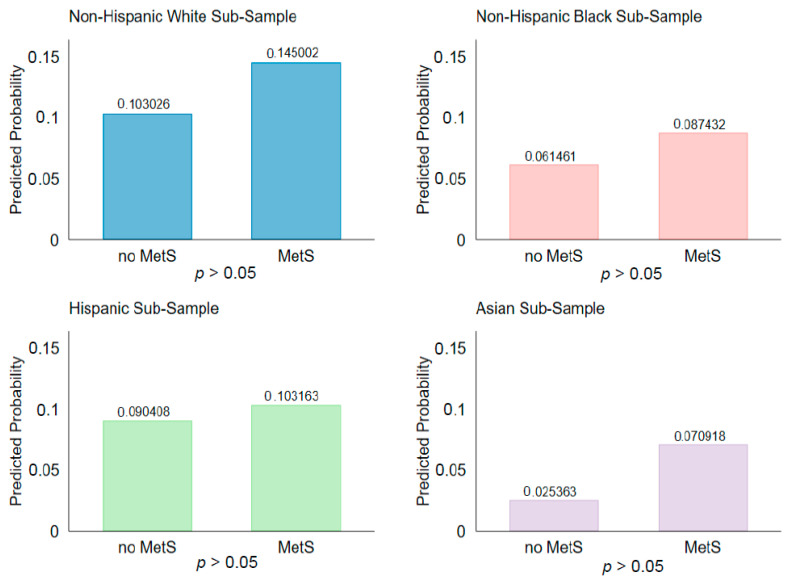
Predicted probability of cancer by metabolic syndrome status, separately for each racial/ethnic Group, NHANES 2011–2018.

All the data analyses were conducted in Stata 16 [34]. First, the descriptive statistics of MetS and cancer prevalence, sociodemographic characteristics, and modifiable lifestyle behaviors of each racial/ethnic group were examined (Table 1). Chi-square tests for categorical variables and linear regression for continuous variables were conducted to examine differences across racial/ethnic groups (Table 1). Binary logistic regression was conducted to examine whether cancer was significantly associated with MetS, with the sociodemographic variables and modifiable lifestyle behaviors accounted for separately for each racial/ethnic group in three steps. First, MetS status was regressed on cancer risk for each racial/ethnic group. Then, the three sociodemographic characteristics (age, gender, and education level) were added into the regression models. In the final step, the three modifiable lifestyle behaviors (tobacco use, alcohol use, and sleep duration) were added into the regression model. The regression results generated in the final models are presented for each group (Table 2). The *mgen* command [35] in Stata was used to compute the predicted probabilities of cancer by MetS status (yes/no) for each of the four racial/ethnic groups (Figure 1). The appropriate sample weights were applied according to the National Center for Health Statistics guidelines to account for the complex survey design, the oversampling of Asian Americans, and taking into account that data on fasting glucose were collected on a subsample of the population [36]. The *svy* command in Stata was used to apply the weights. A *p*-value of 0.05 or below was considered statistically significant.

## 3. Results

The analysis sample consists of 10,220 individuals, including 3604 non-Hispanic white (NHW), 2382 non-Hispanic Black (NHB), 2646 Hispanic/Latino, and 1588 Asian Americans. NHWs had the highest cancer prevalence rate (4.52%), while Asian Americans had the lowest prevalence rate (0.72%). MetS prevalence was highest among Hispanics (31.44%). Socio-demographic characteristics and modifiable lifestyle behaviors varied significantly across racial/ethnic groups (*p* < 0.05). Specifically, tobacco use was high among NHWs (43.21%), while sleep restriction rate (<6 h per day) was particularly high among NHBs (18.48%).

In the first step of the regression analysis where only MetS status was in the logistic regression model, MetS was significantly associated with cancer risk in NHWs (OR = 1.79, *p* = 0.003), NHBs (OR = 2.43, *p* = 0.031), and Hispanics (OR = 1.82, *p* = 0.05); however, not for Asian Americans. When socio-demographic characteristics were added to the models in the second step, the MetS–cancer link was significant only for NHWs (results available upon request). Multivariate logistic regression results were presented in Table 2. Among NHWs, MetS was significantly associated with cancer (odds ratio (OR) = 1.57; 95% confidence interval (CI) = 1.01–2.45), with the covariates held constant. The MetS–cancer association was not statistically significant in the other three racial/ethnic groups.

With regard to modifiable lifestyle behaviors, tobacco use was found to be significantly associated with a higher MetS risk in NHWs (OR = 1.48, 95% CI = 1.00–2.19) and Hispanics (OR = 2.39, 95% CI = 1.09–5.24). Having six or more hours of sleep per day was significantly associated with a lower risk of MetS (OR = 0.15, 95% CI = 0.04–0.54) in Asian Americans. Furthermore, older age was significantly related to higher MetS risks in all but NHBs. Female gender was significantly associated with higher MetS in NHWs, Hispanics, and Asian Americans; however, not in NHBs.

Using the results from the multivariate logistic regression analyses, the predicted probability of cancer by MetS status was computed for the four racial/ethnic groups separately, with all the covariates in the regression model set at their mean values within the entire study sample (Figure 1). For all four groups, the predicted probabilities of cancer were higher among those who had MetS than those without. However, such difference was only statistically significant among the NHW subsample (14.50% vs. 10.30%). Though not statistically significant, a notable difference in the predicted probabilities of cancer by MetS status was observed in Asian Americans (7.09% vs. 2.54%).

## 4. Discussion

While the MetS–cancer link has been receiving growing attention in the past two decades, few studies have examined how the link might vary across racial/ethnic groups or among adults under age 50. This study has four main findings. First, MetS was found to be significantly associated with cancer risk in NHW adults under age 50. While this finding adds to the growing body of literature on the MetS–cancer link [6,12,37,38] in the general population, it was important to note that NHWs were the only group where this association was significant. Previous findings on the relationships between racial/ethnic differences in the MetS–cancer link have been scarce and nuanced. For example, Monestime and colleagues [19] analyzed NAHENS data from 1999 to 2014 and found that among those with MetS, NHB and Hispanic participants were more likely than NHWs to have been diagnosed with obesity-related cancer. The findings indicate a stronger link between MetS and cancer among NHB and Hispanic populations than NHW populations.

Second, the lack of a significant link between MetS and cancer for the other three racial/ethnic minority groups did not necessarily indicate a lack of association. There have been studies that linked MetS and related cardiometabolic conditions, such as diabetes and hypertension, to breast cancer incidence and mortality among African American women [39,40], endometrial cancer in Hispanic women [41], and colorectal and liver cancer in Asian populations [42]. It was possible that in the present study, the limited sample size, particularly given the small number of cancer cases in the sample, resulted in the lack of statistically significant results in the NHB, Hispanic/Latin, and Asian American groups.

Third, modifiable lifestyle behaviors were found to have differential associations with MetS in the four racial/ethnic groups examined. Specifically, tobacco use was a significant correlate of cancer in the NHW and Hispanic groups, but not in the other groups. While the stepwise regression results did not directly indicate any mediating effects of tobacco use, its associations with cancer among different racial/ethnic groups were nonetheless meaningful. In particular, they shed light on the potentially unique physiological and socio-behavioral mechanisms of cancer for each racial/ethnic group. The unique way tobacco use influenced cancer risk in the NHW and Hispanic populations warrants further investigation, especially in the areas of basic science and behavioral studies. It would also be important to explore how tobacco use is linked to MetS, and its potential mediating effects on the link between MetS and cancer. 

Finally, short sleep duration was found to be significantly associated with higher cancer risk; however, this was only in Asian participants, confirming results of a previous categorical meta-analysis study [43]. Although the exact mechanism remains unknown, researchers have suggested several possible pathways that might explain this association and its uniqueness in the Asian American populations. One of the key mechanisms involves metabolic pathways related to obesity, a known cancer risk factor [44]. Sleep restriction potentially leads to altered glucose metabolism, upregulation of appetite, and decreased energy expenditure; in turn, leading to obesity and subsequently, cancer [44]. On top of this, individuals of Asian descent are affected disproportionately with a high burden and unique patterns of cardiometabolic diseases [45,46]. Particularly noteworthy is the “lean yet metabolically unhealthy” phenotype in this population [47,48], which refers to individuals who have a normal body mass index (BMI), yet are affected by metabolic dysfunction. Researchers have suggested that this phenotype is associated with the accumulation of fat in the intra-abdominal area and the liver, combined with insulin resistance [48], factors that have been linked to sleep deficiencies or sleep disorders [49,50,51]. Why sleep is a particularly important factor in metabolic pathways to cancer remains uncertain.

This study is not without limitations. First, the use of self-reported data on cancer and lifestyle behaviors might lead to potential bias. Second, given that the NHANES data used in this study were cross-sectional, our findings do not have causal inferences. Third, previous studies have suggested that obesity-related cancers, such as cancer of the kidney, endometrium, ovary, liver, pancreas, and colorectum, might have more direct associations with metabolic dysfunctions [6,12,19]. Because all cancer types were examined in this study, a link between MetS and a specific type of cancer could neither be identified, nor could any potential racial/ethnic disparities.

Despite the limitations, the findings of the present study shed light on variations in the MetS–cancer link and potential behavioral pathways across four racial/ethnic groups. Future research should leverage existing longitudinal data, such as the surveillance, epidemiology, and end results (SEER)-Medicare linked data, or should collect data from cohort or case-control studies to better examine the causal link between MetS and cancer. Adults under the age of 50, and those in racial/ethnic minorities and other marginalized groups should also be included in further research. 

## Figures and Tables

**Table 1:** Weighted Descriptive Statistics by Race/Ethnicity, NHANES 2011–2016 (N = 10,220).

% of Mean (95% Confidence Interval)	Non-Hispanic White (NHW) (N = 3604)	Non-Hispanic Black (NHB) (N = 2382)	Hispanic (N = 2646)	Asian (N = 1588)	*p*
Cancer (yes %)	4.52% (3.91–5.60%)	1.15% (0.70–1.51%)	2.04% (1.31–2.29%)	0.72% (0.22–1.11%)	<0.001
MetS (yes %)	25.65% (23.23–28.82%)	19.32% (18.49–22.21%)	31.44% (29.65–33.88%)	22.81% (19.69–24.05%)	<0.001
Age, mean	33.89 (33.29–34.50)	32.77 (32.00–33.54)	32.72 (32.31–33.13)	34.15 (33.36–34.94)	<0.001 ^†^
Sex, female	49.70% (48.19–51.17%)	54.52% (53.39–56.84%)	49.58% (48.07–51.16%)	52.30% (50.73–54.70%)	0.0013
Education					<0.001
<HS	9.15% (7.64–10.93%)	14.61% (12.99–16.39%)	33.69% (30.98–36.52%)	8.14% (6.33–10.40%)	
HS or some college	55.78% (52.64–58.86%)	66.14% (63.80–68.41%)	53.81% (51.53–56.08%)	34.75% (30.31–39.47%)	
College or above	35.06% (30.82–28.80%)	19.25% (15.55–21.77%)	12.49% (10.25–14.57%)	57.11% (53.43–62.72%)	
Excessive drinking (yes %)	55.53% (60.47–66.11%)	49.38% (62.19–68.33%)	62.61% (70.62–75.31%)	32.65% (55.76–63.83%)	<0.001
Tobacco use (yes %)	43.21% (39.90–45.46%)	30.16% (27.90–33.45%)	30.65% (28.54–32.77%)	19.18% (18.18–22.38%)	<0.001
Sleep duration					<0.001
<6 h/day	8.85% (8.17–11.26%)	18.48% (17.15–21.21%)	10.38% (9.11–12.46%)	6.50% (5.05–8.43%)	
≥6 h	91.95% (89.69–92.94%)	81.52% (79.82–83.10%)	89.62% (88.00–91.05%)	93.50% (91.86–94.83%)	

^†^*p*-value from regression. Other *p*-values in the column from chi-square test. Abbreviations: MetS = metabolic syndrome; HS = high school.

**Table 2:** Binary logistic regression results of the association between cancer and MetS with covariates, NHANES 2011–2018.

	Non-Hispanic White (NHW)	Non-Hispanic Black (NHB)	Hispanic	Asian
	Odds Ratio (95% Confident Interval)			
MetS	1.57 * (1.01–2.45)	1.57 (0.54–4.60)	1.20 (0.56–2.60)	3.67 (0.70–19.10)
Age, mean	1.06 *** (1.04–1.09)	1.10 ** (1.04–1.16)	1.08 ** (1.03–1.14)	1.08 (0.95–1.24)
Female sex (ref: male)	2.40 *** (1.52–3.79)	1.56 (0.52–4.73)	4.67 *** (2.14–10.19)	4.46 * (1.05–18.88)
College degree (ref: < HS)				
HS or some college	0.96 (0.50–1.86)	0.71 (0.22–2.22)	1.20 (0.50–2.88)	4.48 (0.06–3.72)
College or above	1.41 (0.69–2.90)	1.20 (0.32–4.43)	0.66 (0.21–2.06)	1.07 (0.22–5.33)
Excessive drinking (ref: no)	0.99 (0.60–1.63)	0.96 (0.41–2.24)	0.78 (0.39–1.58)	0.38 (0.05–2.63)
Tobacco use (ref: no)	1.48 * (1.00–2.19)	1.47 (0.52–4.20)	2.39 * (1.09–5.24)	2.09 (0.44–9.84)
Sleep duration ≥ 6 h (ref: < 6 h)	0.83 (0.53–1.32)	0.44 (0.16–1.26)	0.91 (0.23–3.60)	0.15 ** (0.04–0.54)
Constant	0.002	0.0005	0.0003	0.005

* p < 0.05; ** *p* < 0.01; *** *p* < 0.001. Abbreviation: CI = confidence interval; ref = reference group; df = degree of freedom. Abbreviations: MetS = metabolic syndrome; HS = high school.

## Data Availability

Publicly available datasets were analyzed in this study. These data can be found here https://www.cdc.gov/nchs/nhanes/index.htm (accessed on 21 January 2021).
